# The Best Tank for Disinfecting Linen

**Published:** 1887-02-12

**Authors:** 


### THE BEST TANK FOR DISINFECTING LINEN.

"Sanitary Inspector" writes from Manchester:—"Will any reader of The Hospital who has had experience in infectious hospitals kindly inform me if an ordinary slate tank is suitable for being used for carbolic acid, for disinfecting linen from wards and patients? Is the dilute acid found from experience to eat away the slate?

*** Carbolic acid of the strength commonly used for disinfecting purposes does not seem to have any injurious action on slate. A slate tank was so used for many years at the London Fever Hospital without any apparent injury. Slate, however, is apt to get knocked about, and for this reason galvanised wrought iron is preferable.—Ed. T. H.

